# Clinical case of religious delusion in a combination of schizoaffective disorder and epilepsy

**DOI:** 10.1192/j.eurpsy.2022.1822

**Published:** 2022-09-01

**Authors:** E. Gedevani, G. Kopeiko, O. Borisova, A. Iznak, P. Orekhova

**Affiliations:** 1 FSBSI Mental Health Research Center, Researching Group Of Specific Forms Of Mental Disorders, Moscow, Russian Federation; 2 Federal State Budgetary Scientific Institution «Mental Health Research Center», Researching Group Of Specific Forms Of Mental Disorders, Moscow, Russian Federation; 3 Mental Health Research Centre, Laboratory Of Neurophysiology, Moscow, Russian Federation

**Keywords:** Schizoaffective disorder, combined mental pathology, religious delusion, epilepsy

## Abstract

**Introduction:**

Despite existing observations of religious delusions in epilepsy in classical psychiatric literature, such clinical cases are rare in current practice.

**Objectives:**

To reveal features of disease progression, interference of combined mental pathology, treatment specifics, markers of possible harmful behavior.

**Methods:**

Psychopathological, Multichannel eyes closed resting EEG in interictal period.

**Results:**

Patient N, 39 years old, manifested her illness at age 13 with affective bipolar disorder; phases lasted several months each. From age 19, rare recurrent generalized convulsive paroxysms preceded by an aura; non-convulsive paroxysms were observed. The patient was uncritical of paroxysms and discontinued anticonvulsive therapy. At age 29 and 30 she suffered two psychotic attacks (lasting several weeks) with sensory delusions of meaning, staging, persecution, megalomaniacal ideas of apocalyptic content (ideas that she was responsible for possible outbreak of nuclear war, coming of the Apocalypse, her son was the antichrist). Delusional behavior (tried to take the naked infant out into the cold, throwing him out of the window). Anticonvulsive therapy accompanied by antipsychotic medications. Schizoaffective disorder and epilepsy diagnosed. From age 35, acute psychotic attacks with apocalyptic delirium preceded by the same aura lasted maximum one day, followed by partial amnesia. Epileptiform polyspikes (up to 150 μV) registered in the right temporal-central EEG leads.

**Conclusions:**

Presence of religious delusion in combined schizoaffective disorder and epilepsy, requires special approach: combination of anticonvulsants and antipsychotics. Religiosity of patient should be taken into account as well.

**Disclosure:**

No significant relationships.

